# The curvature of cucumber fruits is associated with spatial variation in auxin accumulation and expression of a YUCCA biosynthesis gene

**DOI:** 10.1038/s41438-020-00354-5

**Published:** 2020-09-01

**Authors:** Shengnan Li, Chunhua Wang, Xiuyan Zhou, Dong Liu, Chunhong Liu, Jie Luan, Zhiwei Qin, Ming Xin

**Affiliations:** 1 College of Horticulture and Landscape Architecture, Key Laboratory of Biology and Genetic Improvement of Horticultural Crops (Northeast Region), Northeast Agricultural University, Harbin, 150030 China; 2 College of Horticulture, Fujian Provincial Key Laboratory of Haixia Applied Plant Systems Biology, Fujian Agriculture and Forestry University, Fuzhou, 350002 China

**Keywords:** Plant molecular biology, Plant hormones

## Abstract

Fruit curving lowers the commercial value of cucumber and leads to significant economic losses. The mechanism driving the abnormal curving of cucumber is largely unknown. Through our previous work, we discovered that 2 days post-anthesis (DPA) was the key time point at which various phenotypic and genotypic characteristics of cucumber fruits are determined. Here, we analyzed the transcriptome of the concave (C1) and convex (C2) sides of curved fruits at 2 DPA by Gene Ontology (GO) enrichment and functional pathway enrichment analyses and identified auxin as a putative factor influencing fruit curvature. Changes in the curve angle in the fruits and exogenous auxin treatment analyses showed that asymmetric auxin distribution induces fruit curving. Identification of differentially expressed genes (DEGs) related to auxin and qPCR validation showed that *CsYUC10b* had the most significant differential expression when both sides of the curved fruits were compared. Gene functional analysis showed that the transcript levels of *CsYUC10b* and the auxin concentration were even on both sides of the fruit in *CsYUC10b*-overexpressing plants, which in turn contributed to an equal rate of growth of both sides of cucumber fruits and resulted in a straight shape of the fruits. Thus, we conclude that *CsYUC10b* promotes the formation of straight cucumber fruits, with possible applications in the production and breeding of cucumber.

## Introduction

Fruit shape is a critical factor determining commercial production, germplasm accession classification, and consumer preference. Cucumber (*Cucumis sativus* L.) is an economically important vegetable species of the family Cucurbitaceae and produces both straight and curved fruits. However, fruit curving severely reduces its commercial and economic value^1^. Cucumber fruit shape is controlled by multiple genes and therefore exhibits quantitative inheritance^2^. In addition, different genotypic varieties of cucumber differ significantly in terms of the proportion of curved fruits versus straight fruits^3^. However, little is known about the mechanisms underlying fruit curving, which is a crucial factor for developing new varieties.

The growth of cucumber fruit can be separated into cell division, fruit elongation, expansion, and ripening stages^4^. The fruit shape obtained at the end of this process depends on the ovary shape and is controlled by preanthesis processes^5^. In addition, several hormones regulate fruit elongation and shape in response to environmental stimuli^6^. For instance, the endogenous level of auxin is closely associated with fruit shape, set, and size^7^. Downregulation of *auxin response factor 7* (*SlARF7*) in tomato (*Solanum lycopersicum*) results in heart-shaped fruits with a thick pericarp^8^. Auxin homeostasis depends on the balance between its biosynthesis, metabolism, and inter- and intracellular transport^9^. In *Arabidopsis thaliana* and maize (*Zea mays*), auxin biosynthesis involves a two-step pathway starting with the conversion of tryptophan to indole-3-pyruvate (IPA) by tryptophan aminotransferase (TAR2), a member of the aminotransferase Arabidopsis (TAA) family, followed by catalysis of IPA into the major endogenous auxin indole-3-acetic acid (IAA) by YUCCA flavin-containing monooxygenases^10^. In addition, weak insensitive 2 (WEI2) and weak ethylene insensitive 7 (WEI7) are known rate-limiting enzymes involved in the biosynthesis of the auxin precursor tryptophan. Loss of *WEI2* and *WEI7* has no obvious effect on auxin phenotypes; however, upregulation of *WEI2* and *WEI7* contributes to auxin accumulation in the roots^11^. The spatiotemporal distribution of auxin is controlled by auxin resistant 1 (AUX1), like aux (LAX1-3) proteins mediate auxin influx, and PIN-formed (PIN1-8) mediates auxin efflux, all of which collectively regulate organ-level auxin transport^12,13^.

Auxin plays an important role in apical hook development in *A. thaliana* by inducing differential cell growth on both sides of the hook. Higher auxin concentration on the inner side of the hook compared with the outer side correlates to reduced cell elongation^14^. In addition, the auxin biosynthesis genes *YUCCA1*, *TAA1*/*WEI8*, and *TAR2* are upregulated in the hook region, and the *wei8*, *tar2*, and *yuc1*/*2*/*4*/*6* mutants of *A. thaliana* display improper apical hook formation^15,16^. Furthermore, the auxin transporter genes AUX1 and LAX3 and auxin response factors (ARFs) also play an essential role in differential hook growth^17^. Ethylene enhances auxin biosynthesis in the inner side of the hook by upregulating *TAR2*, *IAA3*, *IAA12*, and *IAA13*, resulting in differential cell growth. This hormone also stimulates hypocotyl elongation upon light exposure by activating IAA and upregulating *TAA1*, *YUCCA1*, and *YUCCA5* as well as the auxin transport-related genes *AUX1*, *PGP19*, *PIN3*, and *PGP1*^18^. In a previous study, we analyzed the transcriptomes of curved cucumber fruits retrieved from the expressed sequence tag database^19^ and identified a putative auxin synthesis gene, *CsYUC10b* (*Csa3G190380*), that was likely associated with fruit curvature. Herein, we found that *CsYUC10b* induced the formation of straight fruit by promoting consistent bilateral auxin biosynthesis in the fruits.

## Materials and methods

### Plants

Cucumber varieties L18, D0328-3, and D0859, with different genotypes and respective fruit curving ratios of 52.56, 39.28, and 28.21, were selected for the study^20^. The seeds of late-generation inbred lines from our laboratory were sown in pots containing a 1:1 mixture of soil and substrate. For the L18, D0328-3, and D0859 genotypes, 150, 50, and 50 plants were grown, respectively, in a controlled chamber at Northeast Agricultural University under a 12-h photoperiod at 29 °C/17 °C (day/night) and 75% relative humidity. The flowers were manually pollinated. L18 produced 3–4 fruits per plant, and a total of 580 fruits, including 308 curved fruits and 272 straight fruits, were used in this study. The number of fruits produced by the D0328-3 and D0859 genotypes was 220 (of which 86 were curved) and 200 (of which 56 were curved), respectively. The detailed data are shown in Supplementary Table S1. The nodes of 8–15 fruits from five individual plants showing consistent growth at 2, 4, 6, 8, and 10 days post-anthesis (DPA) were collected, flash-frozen in liquid nitrogen, and stored at −80 °C. The L18 T_2_ generation inbred lines OX4, OX7, and OX10 were used for transgenic experiments, and 50 plants of each line were grown under similar conditions.

### Library construction and transcriptome analysis

A study by Wang et al.^21^ showed that 2 DPA was a key time point to determine whether cucumber fruits would become curved or straight. A transcriptome expression profile analysis of curved (including the concave and convex sides) and straight fruits at 2 DPA showed that upregulated genes on the convex side were important determinants of cucumber fruit curvature^21^. Consequently, in this study, we focused on the difference between the concave (C1) and convex (C2) sides of the curved fruits at 2 DPA. The exocarps (0.1 cm thick) were dissected from the C1 and C2 sides of nine curved fruits of the L18 variety at 2 DPA. These exocarps were used for RNA extraction using TRIzol reagent (Invitrogen, Carlsbad, CA, USA). The quality and quantity of the purified RNA were then determined by measuring the absorbance at 260 nm/280 nm (A260/A280) using a SmartSpec Plus instrument (BioRad, USA). The RNA integrity was further verified by 1.5% agarose gel electrophoresis. For each sample, 3 g of total RNA was used for RNA-seq library preparation. The libraries were prepared according to the manufacturer’s instructions and subjected to an Illumina GAIIx system for 80 nt single-end sequencing by ABlife, Inc. (Wuhan, China) or to a HiSeq 2000 system for 100 nt pair-end sequencing by BGI, Inc. (Shenzhen, China).

Information in the Sequence Read Archive database (accession number: SRP111902) was used for transcriptome analysis^21^. After trimming the adaptor sequences, we obtained clean raw reads from the raw sequence reads, which were filtered based on base quality (*Q* ≥ 20)^22^. All the clean reads were aligned to the reference genome (http://cucurbitgenomics.org/) of cucumber (*Chinese Long genome V2*) by TopHat2 (http://tophat.cbcb.umd.edu/). All the values were expressed in reads per kilobase pair per million reads (RPKM) based on the read and gene locations in the genome. Differentially expressed genes (DEGs) were chosen based on the following criteria: *p* value < 0.01, |log2 ratio ≥ 1| and false discovery rate (FDR) < 0.05. Gene Ontology (GO) enrichment analysis of the DEGs was implemented by the agriGO package (http://bioinfo.cau.edu.cn/agriGO/index.php). An enrichment score (ES) of ≥1.3 and *p* < 0.05 was considered significant^23^. Gene set enrichment analysis (GSEA) was used to analyze gene sets and biological pathways by KOBAS 3.0^24^.

### Real‑time quantitative PCR

The exocarps of both sides of L18 curved fruits at 0–10 DPA were used to verify the transcriptomic results, and samples of roots, stems, leaves, stem apices, female flowers (blooming), and male flowers (blooming) were also measured. Fruits were sampled from L18 to analyze the expression of *CsYUC10b*. Total RNA was isolated using TRIzol reagent (Invitrogen) according to the manufacturer’s instructions and reverse transcribed to cDNA using a kit (Toyobo, Japan). qRT-PCR was performed using 10 µL of 2x Fast qPCR Master Mixture (DiNing, China), 0.5 μL each of the forward and reverse primers (10 μM), 2 μL of cDNA template and ddH_2_O such that the total volume was 20 μL. The reaction program was as follows: initial denaturation at 95 °C for 3 min; by 40 cycles of denaturation at 95 °C for 10 s, annealing at 56 °C for 30 s and elongation at 72 °C for 30 s; and a final extension at 72 °C. The fluorescence signals were analyzed using qTOWER 2.0 (AnalytikJena, Germany). The relative expression of the candidate genes relative to the internal control *EF1a*^25^ was calculated using the 2^−ΔΔCT^ method^26^. Three biological and technical replicates were analyzed. The primers used are listed in Supplementary Table S2.

### Subcellular localization

The coding sequences of *CsYUC10b* excluding termination codons (TAG) were amplified by PCR, cleaved with *Hind*III and *Bam*HI, and then cloned into a pGII-EGFP expression plasmid using T4 DNA ligase (Invitrogen)^27^. The recombinant 35S:CsYUC10b-GFP plasmid and empty plasmids were transformed into isolated protoplasts from *A. thaliana*^28^, which were then observed under a confocal spectral microscope equipped with 488 and 580 nm filters (Leica, Germany).

### Generation of *CsYUC10b* transgenic cucumber

The amplified *CsYUC10b* sequence was cloned into a p1250 vector harboring a glyphosate resistance gene^29^ under a constitutive promoter following *Xcm*I digestion and T_4_ ligation (Invitrogen). The p1250-*CsYUC10b* and empty plasmids were transformed into *Agrobacterium* LBA4404, which was then used to infect the cotyledons of L18 plants^30^. The cloned cotyledons were regenerated as described previously^31^, and the transgenic lines were screened in MS media supplemented with 1 mg/L glufosinate^32^. T_1_ and T_2_ plants were identified by PCR and qRT-PCR using specific primers (Supplementary Table S2). Samples were collected from five individual plants for each biological repeat, and three biological and technical repeats were tested. The change in the curve angle was observed from 0 to 12 DPA between the *CsYUC10b* overexpression fruits and the control group fruits. The exocarps were collected from both sides of the fruits at 4 DPA to analyze the auxin content and the *CsYUC10b* expression levels.

### Treatment with 1-naphthaleneacetic acid (NAA), 1-naphthoxyacetic acid (NOA) and aminoethoxyvinylglycine (AVG)

The ovaries of the straight and curved L18 fruits were sprayed at preanthesis (0 DPA) on one side (the concave side for the curved fruit) with 0.1, 0.15, and 0.20 µM NAA (a synthetic auxin); 0.1 µM AVG (an auxin biosynthesis inhibitor); or 0.1 µM NOA (an auxin transport inhibitor). At the same time, both sides of the curved fruits were sprayed with the abovementioned solutions. The controls were sprayed with the same volume of water (10% Tween was used as an adsorbent). The NAA stock was 5 mmol/L, and the working volumes of NAA used were 20, 30, and 40 µL, with 10% Tween. Similarly, the volume of NOA and AVG was 20 µL, with 10% Tween. We sprayed the solutions using an even-bore squirt bottle, and each fruit was treated only once. The exocarps were collected from both sides of the suitably treated fruits at 4 DPA to analyze the auxin content and transcript levels of *CsYUC10b*. The change in the curve angle was observed from 0 to 12 DPA.

### Determination of auxin content

The exocarp was collected from both sides of straight and curved fruits of the transgenic and control L18 plants at 0, 2, 4, 6, 8, and 10 DPA. Auxin was extracted according to the protocol of Weiler^33^ and measured using an ELISA kit specific for IAA (Meimian Industrial Co., Ltd, China). Six replicates were tested per sample, and the experiment was performed three times. The IAA levels in the different groups were compared by Student’s *t*-tests (*p* < 0.05).

## Results

### Transcriptome analysis of curved cucumber fruits

**Fig. 1:**
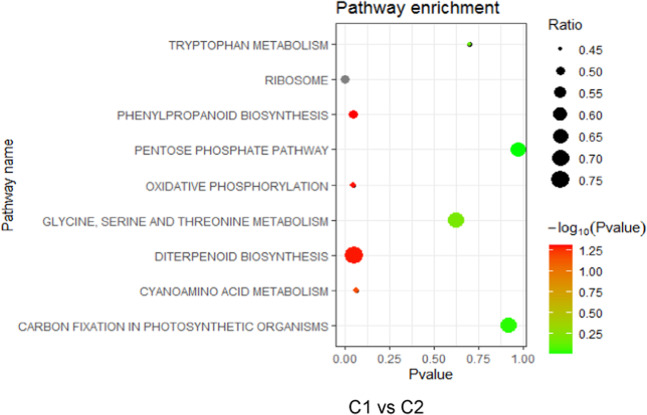
Gene Ontology (GO) enrichement analysis of specific DEGs. Functional pathway enrichment on the concave (C1) and convex (C2) sides of curved fruits at 2 DPA according to GSEA

Transcriptome analysis revealed 27.07 and 20.59 million raw reads from C1 (clean reads, 85.71%) and C2 (clean reads, 85.71%) after low-quality adaptor and barcode sequences were removed (Supplementary Table S3). The sequencing quality analysis, sequence content distribution, and length distribution of the clean reads indicated good sequencing quality by RNA-seq (Supplementary Fig. S1). A total of 4313 DEGs were identified between the C1 (convex) and C2 (concave) sides of the curved fruits, of which 2351 were upregulated and 1962 were downregulated in C1 relative to C2 (Supplementary Fig. S2). Using ES ≥ 1.3 and *p* value < 0.05 as criteria, 15 categories of significantly enriched genes were identified by GO analysis in C2, including 9 related to photosynthesis, adaxial/abaxial formation, redox processes and responses to auxin stimuli (Supplementary Table S4), suggesting the possible involvement of light in fruit curving. Likewise, the C1-specific categories were enriched in protein kinase activity, the ethylene-mediated signal pathway, regulation of cell size, regulation of cell shape, and polar auxin transport (PAT). In addition, several of the identified DEGs were related to plant hormones, including ethylene, auxin, cytokinin, abscisic acid, and gibberellins. The ethylene signal transduction-related genes were expressed predominantly in the fruits, followed by the genes coding for auxin production (Supplementary Table S5). In a previous report, ethylene was found to promote cucumber fruit curving^21^. Further functional pathway enrichment analysis showed that tryptophan metabolism, ribosomes, phenylpropanoid biosynthesis, and six other pathways had significant enrichment (Fig. 1). Importantly, tryptophan was the major auxin biosynthetic precursor, as inferred through our experiments. Thus, we surmised that auxin is also involved in fruit curving.

### Asymmetric auxin levels are correlated with cucumber fruit curving

**Fig. 2:**
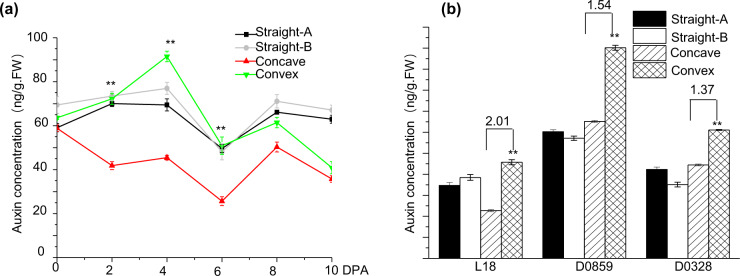
Temporal changes in auxin concentration in cucumber fruits. **a** Auxin concentration on the C1 and C2 sides of curved fruits and on both sides of straight fruits at 2, 4, 6, 8, and 10 DPA. **b** Auxin concentration on the C1 and C2 sides of curved fruits and on both sides of straight fruits of the L18 (52.56%), D0859 (39.28%) and D0328-3 (28.21%) genotypes at 4 DPA. The data are the means (±SEs) of three independent experiments, with five replicates each (*p* < 0.05)

The curve angle of the L18 fruits differed between 0–20°, 20–60°, and ≥60° during development from 0 to 18 DPA. The increase was gradual between 0–4 DPA and peaked at 4–7 DPA, after which the angle decreased slowly from 8 to 18 DPA (Supplementary Fig. S3a). Accordingly, we divided the fruit curving process into the formation, maintenance, and opening phases. A curve angle of 0–20° at 0 DPA correlated to straight fruit, while an angle >20° was indicative of a curved shape (Supplementary Fig. S3b), indicating that ovary growth plays a decisive factor in early curving. To determine the role of auxin in the development of curved fruits, we measured auxin levels on both sides of curved and straight fruits from 2 to 8 DPA. Auxin levels were markedly higher on the convex side than on the concave side of the curved fruits, peaking at 4 DPA and gradually declining thereafter. In contrast, both sides of the straight fruits had similar auxin levels throughout development (0–10 DPA) (Fig. 2a). The auxin concentration was 2.01-, 1.54- and 1.37-fold higher on the convex side than on the concave side in the fruits of the L18, D0859, and D0328-3 varieties, respectively, at 4 DPA; these results correlate with the respective curved fruit ratios of 52.56%, 39.28%, and 28.21% in these genotypic varieties. Not surprisingly, both sides of the straight fruits showed similar auxin concentrations regardless of genotype (Fig. 2b). Overall, asymmetric auxin distribution likely induces cucumber fruit curving through differential growth of the two sides.

### Auxin distribution directly controls fruit curving

**Fig. 3:**
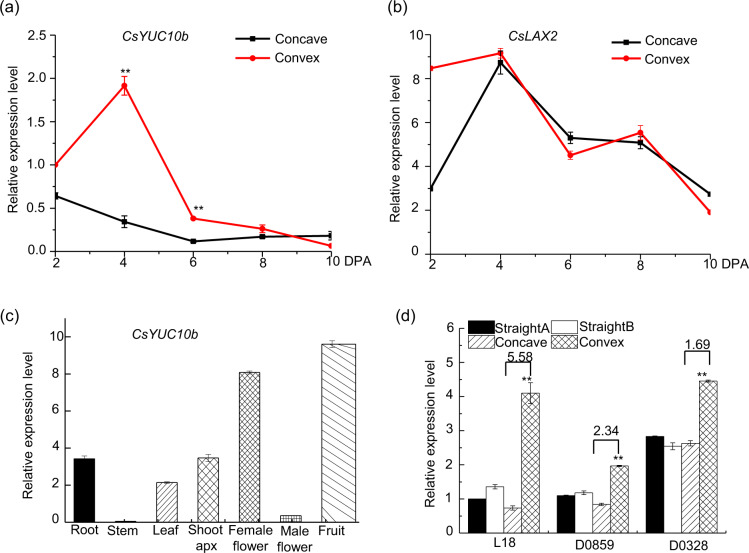
Expression patterns of auxin-related genes in cucumber. **a** *CsYUC10b* mRNA levels on the concave and convex sides at 2, 4, 6, 8 and 10 DPA. **b** *CsLAX2* mRNA levels on the concave and convex sides at 2, 4, 6, 8, and 10 DPA. **c** *CsYUC10b* levels in different tissues of L18 plants. **d** *CsYUC10b* levels in curved and straight fruits of the L18, D0859 and D0328-3 genotypes at 4 DPA. *CsEF1a* was used as a reference gene. The data are the means (±SEs) of three independent experiments, with five replicates each (*P* < 0.05). *P < 0.05, and **P < 0.01)

Asymmetric auxin distribution during fruit curving is likely due to its aberrant local synthesis and polar transport. To test this hypothesis, we treated one side of the ovaries from straight fruits with synthetic auxin (NAA) at 0 DPA and observed faster growth of the fruit on the treated versus the untreated side, eventually resulting in a curved shape. The maximum curvature (approximately 155°) was observed in fruits treated with 0.2 μM NAA at 6 DPA (Fig. 3a). Interestingly, the application of 1 µM NAA on the concave side of ovaries with a 45° curve decreased the curvature at 4 DPA, and the fruits ultimately had a straight shape (Fig. 3b). Furthermore, when ovaries with 70-90° curving were treated with 0.1, 0.15, and 0.2 μM NAA on the concave side, the higher doses were able to decrease the angle of curvature at 2 DPA; however, while 0.15 µM NAA restored the straight shape of the final fruit, 0.2 µM NAA resulted in a backward curvature (Fig. 3c). Through further analysis, we found that in response to the application of 0.15 μM NAA, the auxin content was consistent on both sides of fruits at 4 DPA (Fig. 3d). Consistent with these findings, the application of the auxin transport inhibitor NOA to both sides of curved ovaries decreased the angle of curvature (Fig. 3e), as well as the differential bilateral auxin levels (Fig. 2f), compared to that of the untreated control at 4 DPA. To further support our conclusions, we examined the impact of the auxin biosynthesis inhibitor AVG). Following the application of AVG (0.1 μM) to the concave side of the ovary during the preanthesis stage, we observed that the fruit exhibited a relatively straight phenotype at 4 DPA (Supplementary Fig. S4). This approach did not achieve the desired effect because both ethylene and auxin synthesis were inhibited by AVG, and ethylene has been reported to contribute to cucumber fruit curving^21^. Overall, asymmetric auxin distribution led to uneven growth of both sides of the fruit, resulting in the formation of curved fruits.

### *CsYUC10b* plays a crucial role in fruit curving

**Fig. 4:**
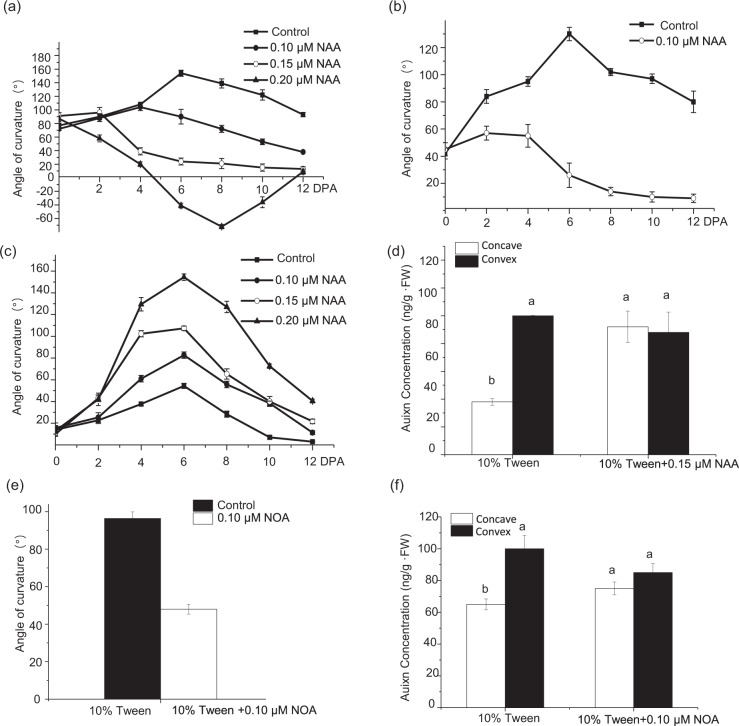
Effects of exogenous auxin on cucumber fruits. **a** Angle of curvature of straight fruits treated with 0.1, 0.15, and 0.2 µM NAA at 2, 4, 6, 8, and 10 DPA. **b** Angle of curvature of 45° curved fruits treated with 0.1 µM NAA on the concave side at 2, 4, 6, 8, and 10 DPA. **c** Angle of curvature of 70–90° curved fruits treated with 0.1, 0.15, and 0.2 µM NAA on the concave side at 2, 4, 6, 8, and 10 DPA. **d** Auxin concentration on the concave and convex sides of the control and 0.1 µM NAA-treated fruits at 4 DPA. **e** Angle of curvature of curved fruits treated with 0.1µM NOA on both sides at 4 DPA. **f** Auxin concentration on the concave and convex sides of control and NOA-treated curved fruits at 4 DPA. *CsEF1a* was used as a reference gene. The data are the means (±SEs) of three independent experiments, with five replicates each (*p* < 0.05)

We identified 33 auxin homeostasis-related genes from the transcriptome data of curved fruit exocarps (Table 1), of which *YUC10b* (*Csa3G190380*), *GH3.6* (*Csa3G431430*), *AUX1* (*Csa3G731880*) and *LAX2* (*Csa2G264590*) were differentially expressed between C1 and C2 (|log2 ratio ≥ 1| and *p* value = 0.01), and only *CsYUC10b* and *CsLAX2* were upregulated on the convex side compared to the concave side of the fruits. The transcript levels were measured in the curved fruits from 0 to 10 DPA, and we observed a marked upregulation of *CsYUC10b* on the convex side compared with the concave side at 2–8 DPA, which was consistent with the maximum curvature recorded at 4 DPA (Fig. 4a). However, *CsLAX2* expression was similar on both sides of the curved fruits from 4 to 10 DPA (Fig. 4b). Thus, we hypothesized that *CsYUC10b* is a key gene involved in cucumber fruit curving. We also analyzed the expression of *CsYUC10b* in all the organs of the L18 plants and detected low levels of transcripts in the male flower buds and stems; moderate levels in the roots, leaves and shoot apices; and increased levels in open female flowers. The highest levels were detected in the fruits (Fig. 4c). Furthermore, the *CsYUC10b* transcript levels were 5.58-, 2.34- and 1.69-fold higher in the convex side compared with the concave side of curved fruits from the L18, D0859, and D0328-3 plants, respectively, at 4 DPA, while no significant differences were detected on either side of the straight fruits (Fig. 4d).

**Table 1:** List of 33 auxin homeostasis-related genes on the C1 and C2 sides of curved cucumber fruits

			RPKM			
Gene ID	Log_2_ (ratio) (C1/C2)	*p* value	Concave (C1)	Convex (C2)	Functional annotation	Gene family
Csa3G133910	0	1	0	0	YUC3	Flavin monooxygenase (YUC)
Csa2G379350	−0.920955361	2.91733E−06	3.82	6.07	YUC4	
Csa1G242600	1.715031143	0.453125	0.29	0.07	YUC6	
Csa2G375750	−2.092323779	0.109375	0.07	0.27	YUC6	
Csa3G619930	0	1	0	0	YUC8	
Csa6G087870	28.11200867	0.0625	0.35	0	YUC8	
Csa6G087880	−0.470835402	0.377085587	2.15	2.51	YUC8	
Csa3G190380	−1.342856524	2.1685E−193	58.36	125.04	YUC10	
Csa7G390100	−0.751286861	0.049800114	1.13	1.65	YUC10	
Csa6G454350	−0.852544726	5.75218E−12	8.23	12.58	ILR1	IAA-leucine resistant 1-like (ILL)
Csa3G778230	0.251630622	0.181175894	7.06	5.01	ILR1	
Csa3G778240	−0.264276462	0.191899854	5.12	5.2	ILR1	
CsaUNG029820	0	1	0	0	ILL2	
Csa1G065960	0.036804848	0.74914208	22.16	18.26	ILR3	
Csa2G423590	−0.886864786	5.311E−54	57.62	90.05	ILR3	
Csa3G415100	−0.014845026	0.917664014	21.01	17.96	ILR3	
Csa3G416140	0.407463355	8.67334E−07	43.24	27.58	ILR3	
Csa6G384060	0.939525087	6.7467E−06	5.06	2.24	ILL6	
Csa3G088930	27.52704617	0.125	0.09	0	GH3.6	Gretchen Hagen 3 (GH3)
Csa3G431430	1.054517609	1.64345E−07	5.48	2.27	GH3.6	
Csa6G125240	25.52704617	1	0.02	0	GH3.6	
Csa4G415930	28.84897427	0.0078125	0.26	0	UGT74D1	UDP-glucosyl transferase (UGT)
Csa6G418940	25.52704617	1	0.06	0	UGT74D1	
Csa1G025070	−0.818359813	1.46901E−39	26.98	40.19	PIN1	PIN-formed (PIN)
Csa1G042820	−0.258996759	0.000400637	22.22	22.48	PIN1	
Csa4G430820	−0.175739787	0.369164706	3.84	3.66	PIN1	
Csa5G576590	0.540204405	1.30289E−05	9.5	5.52	PIN3	
Csa2G074170	0	1	0	0	PIN5	
Csa5G284520	−0.182890989	0.171454885	6.11	5.88	PIN7	
Csa3G827360	−0.5695301	3.64434E−06	9.95	12.5	PIN8	
Csa3G731880	1.731150808	4.41503E−33	11.09	2.85	AUX1	Auxin resistant 1 (AUX1)
Csa2G264590	−3.221606796	1.33316E−20	0.5	3.88	LAX2	
Csa7G010800	0.692825349	4.71397E−08	14.23	7.48	LAX2

### *CsYUC10b* cloning and expression

To further investigate the role of *CsYUC10b* in fruit curving, we analyzed its full-length (1158 bp) CDS (*Csa3M190380.1*), which encodes a putative 385 amino acid protein. As shown in Supplementary Fig. S5a, *CsYUC10b* has a 171 bp 5′ untranslated region (UTR), a 188 bp 3′ UTR, four exons and three introns. Phylogenetic tree analysis (Supplementary Fig. S5b) showed that *CsYUC10b* is orthologous to the *YUC* genes of *Cucumis melo* (*CmYUC10*), *Morus notabilis* (*MnYUC10*), *Theobroma cacao* (*TcYUC10*) and *Glycine soja* (*GsYUC10*), with respective sequence homologies of 90.39%, 64.25%, 61.66% and 58.29% (Supplementary Fig. S5c). The *CsYUC10b* sequence was cloned into an expression vector under the control of the constitutive 35S promoter and expressed in *A. thaliana* protoplasts. As shown in Supplementary Fig. S6, the CsYUC10b-GFP fusion protein was localized in the cytoplasm, whereas the empty plasmid controls showed diffuse fluorescence.

### *CsYUC10b* promotes straight fruit development in cucumber by balancing auxin biosynthesis

**Fig. 5:**
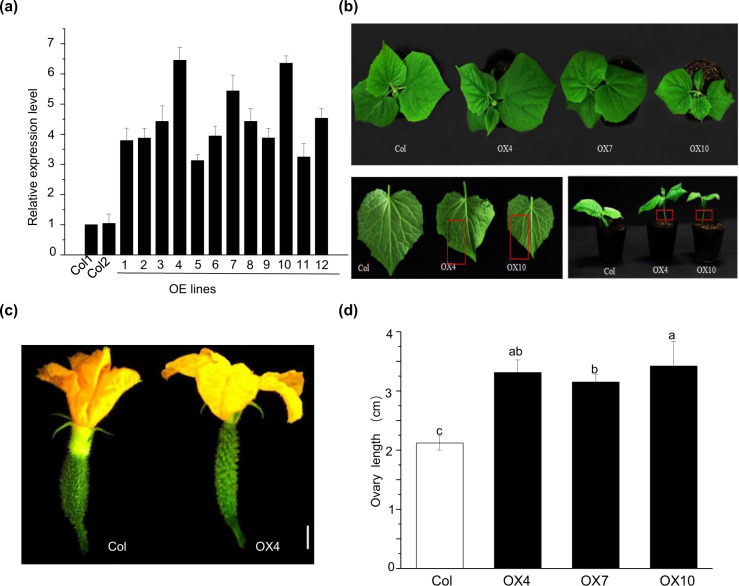
Auxin hyperaccumulation in *CsYUC10b* transgenic plants. **a** *CsYUC10b* levels in the leaves of control (Col) and *CsYUC10b*-overexpressing (OX) plants. **b** Downward curled leaves and stem elongation of Col and OX. **c** Ovary growth in Col and OX plants. **d** Ovary length in Col and OX plants. The data are the means (±SEs) of three independent experiments, with five replicates each. *P < 0.05, and **P < 0.01

**Fig. 6:**
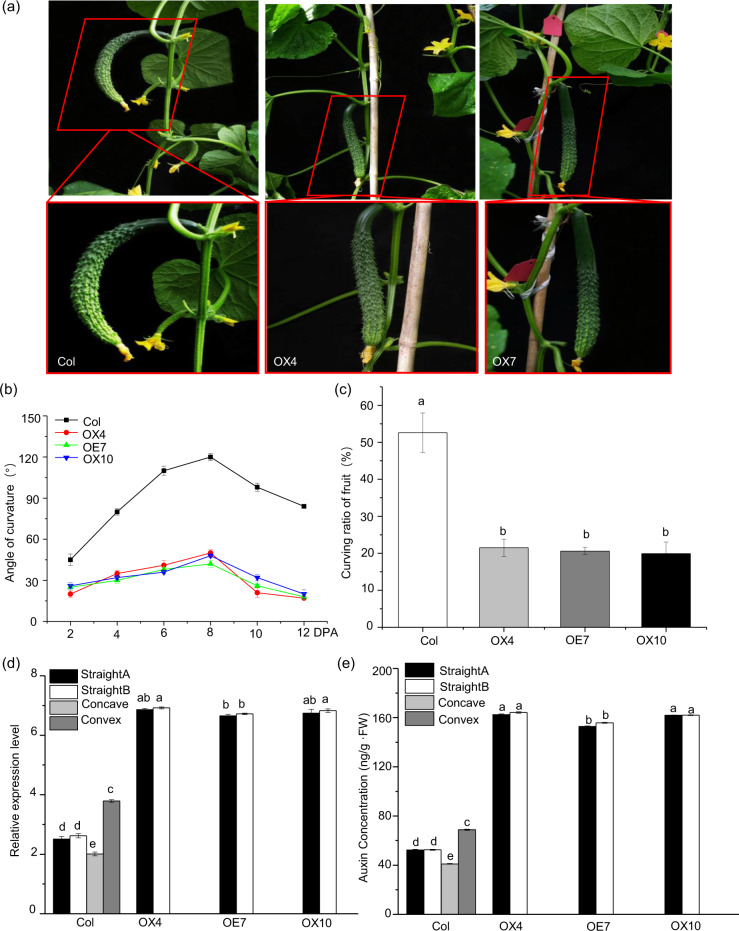
Effects of *CsYUC10b* overexpression in cucumber. **a** Phenotypes of fruits of the control (Col) and *CsYUC10b*-overexpressing (OX) plants at 4 DPA. **b** Angle of fruit curvature in Col and OX plants at 2, 4, 6, 8, and 10 DPA. **c** Percentage of curved fruits of Col and OX plants. **d** *CsYUC10b* levels on both sides of Col and OX fruits. **e** Auxin concentration in Col and OX plants at 4 DPA. The data are the means (±SEs) of three independent experiments, with five replicates each (*P* < 0.05)

Our findings thus far indicated that asymmetric auxin accumulation in cucumber fruits caused uneven growth and eventual curving. To test this hypothesis, we generated *CsYUC10b*-overexpressing transgenic cucumber lines, screened the resistant plants with 1 mg/L glufosinate, and ultimately obtained forty-four resistant transgenic plants. Our PCR confirmation results showed that the whole identified 1800 bp fragment included *CsYUC10b*, which was 1158 bp, and a 600 bp fragment from the p1250 vector (Supplementary Fig. S7). Twelve PCR-positive plants that displayed increased growth were subjected to a qPCR-based analysis, the results of which showed that OX4, OX7, and OX10 expressed the highest levels; these plants were self-pollinated for subsequent studies in the T_2_ generation (Fig. 5a). The transgenic plants displayed phenotypes associated with auxin hyperaccumulation, such as downward curled leaves and rapid stem elongation at the seedling stage. (Fig. 5b). Furthermore, compared with the wild-type controls, the *CsYUC10b* transgenic plants showed faster growth of the ovaries of female flowers (Fig. 5c). As shown in Fig. 4d, the respective ovary lengths in the OX4, OX7, OX10 transgenic plants and control plants were 3.31, 3.15, 3.42, and 2.12 cm. These results strongly indicated that *CsYUC10b* promotes auxin hyperaccumulation and ovary growth. Interestingly, at 4 DPA, the *CsYUC10b*-overexpressing plants produced straight fruits compared with the highly curved (80°) fruits of the control plants (Fig. 6a). Furthermore, the angle of fruit curvature decreased significantly for the transgenic plants compared to the control plants at 0–10 DPA (Fig. 6b). We observed 188, 182, 189, and 176 fruits produced by the control, OX4, OX7, and OX10 lines (Supplementary Table S6). As shown in Fig. 6c, the proportion of curved fruits was only 21.5, 20.6, and 19.9% for the OX4, OX7, and OX10 transgenic lines compared to 52.6% for the control plants (Fig. 6c). In addition, the *CsYUC10b* transcript levels and auxin concentration were significantly higher in the transgenic plants than in the control plants and showed a positive correlation (Fig. 6d, e). Consistent with these findings, the auxin levels were even on both sides of the transgenic fruits, which resulted in a straight shape (Fig. 6a). Overall, *CsYUC10b* induces straight fruit development in cucumber by equilibrating auxin biosynthesis on both sides of the fruit.

## Discussion

Asymmetric auxin distribution is a key factor driving the differential growth of plant organs. For instance, auxin efflux controls apical hook development in *A. thaliana*^34^, and significant auxin accumulation in the inner side of apical hook inhibits bean growth^35^. Furthermore, the application of exogenous auxin or the auxin transport inhibitor 1-naphthylcarbamylbenzoic acid (NPA) impairs apical hook formation^34^. Hormones such as ethylene and gibberellins (GAs) can skew auxin synthesis and polar transport to one side of plant organs and inhibit cell growth while increasing the same on the contralateral side^36^. In this study, we observed temporal changes in auxin levels during cucumber fruit development. In addition, the auxin concentration was slightly higher on the convex side than on the concave side of the curved fruits at 0–2 DPA, and the differential expression peaked at 4–7 DPA, corresponding to increased curvature. As the curvature decreased from 8 to 18 DPA, the auxin levels gradually became even between both sides. Thus, the angle of fruit curvature is a consequence of differential growth controlled by auxin distribution.

The key step in auxin synthesis is YUCCA-mediated conversion of IPA to IAA^37^. Overexpression of *YUC* genes increases auxin production in *A. thaliana*, while the loss of function of a single *YUC* gene does not affect plant growth^38^. However, a previous study showed that mutation at a single *YUC* locus prevented the growth of *A. thaliana* and maize seedlings^39^. Typically, *YUC*-overexpressing lines show phenotypes associated with auxin overproduction. For instance, heterologous expression of *AtYUC6* in potato (*Solanum tuberosum*) plants increased the auxin concentration, resulting in plants with relatively narrow and downward-curling leaves, relatively long petioles, and increased height and longevity^40^. Similarly, *AtYUC6* overexpression in poplar led to rapid shoot growth and slowed the development of the main root, and root hair length increased^41^. In this study, overexpression of *CsYUC10b* in cucumber resulted in downward-curling leaves and elongated stems and ovaries, which supports the involvement of *CsYUC10b* in auxin biosynthesis.

*YUC* genes also regulate auxin biosynthesis during fruit development. The upregulation of *PpYUC11* in peach (*Prunus persica*) is correlated with increased auxin synthesis and fruit maturation^42^. Likewise, *CmYUC6* and *CmYUC11* are significantly upregulated in the seeds and fruit mesocarp of melon (*Cucumis melo*)^43^, and high YUCCA levels in strawberry seeds correlate with auxin biosynthesis and fruit development^44^. *CsYUC11* is expressed in the male flowers of *A. thaliana*, and its overexpression promotes auxin accumulation and elongation of the pedicel and stamen^45^. Consistent with this, overexpression of *CsYUC10b* in cucumber induced symmetric auxin biosynthesis on both sides of the fruits, resulting in the formation of straight fruits.

Fruit development typically progresses from preanthesis ovary growth to ripening^46^. In cucumber, cell division at the earliest stages (0–4 DPA) plays a decisive factor in the final fruit size and shape, and fruit elongation peaks at ~4–12 DPA. This is consistent with the findings that rapid cell enlargement occurs after the frequency of cell division decreases^47^. Furthermore, the number of cells in the ovary is also a major factor determining cell division and fruit development^48^. Increased cell numbers and growth in the longitudinal direction determine ovary length and fruit size in cucumber, respectively^49^. In a previous study, we detected shorter cells on the concave side compared to the convex side of curved cucumber fruit at 6 DPA (Supplementary Fig. S8), indicating that fruit curving depends on the tightly regulated differential growth of cells^20^. Since auxin controls both cell division and cell elongation^50^, auxin-dependent differential growth likely progresses through the different developmental phases as follows: (1) establishment of asymmetric auxin biosynthesis that skews cell growth rates, (2) regulation of the increasing-to- maximum phase transition and the spatial stabilization of auxin distribution, (3) control of the maximum-to-decreasing phase transition, and (4) gradual loss of auxin maxima.

Our previous work involved a quantitative trait locus (QTL) analysis of an F_2_ population derived from a cross between the curved-type inbred line L18 and straight-type inbred line D9320. There were 116 pairs of SSR markers applied to these lines; however, only four markers were detected on a linkage group, and the QTL map distance was 2.5 cM on chromosome (chr) 6^51^. Given the advancement of genome sequencing technology, RNA sequencing (RNA-seq) has emerged as a comprehensive and accurate tool for analyzing key genes and molecular mechanisms. In this study, we used RNA-seq to identify auxin involvement in fruit curving and screened a candidate gene, *CsYUC10b* (*Csa3M190380.1*), located on chr 3. Through exogenous auxin (NAA, NOA, and AVG) treatment and gene functional verification, we found that overexpression of *CsYUC10b* promotes the development of straight fruit in cucumber. These results do not account for *CsYUC10b*, which had no relationship with major effector genes responsible for fruit curving. In our future studies, we propose to utilize the genomic sequences, develop additional markers for QTL fine mapping and identify major genes responsible for fruit curving. This would help us explore the relationship between auxin-coding genes and the other major signaling sequences.

## Supplementary information

Editorial Certificate

Supplementary Information
