# Supplementary material for: The curvature of cucumber fruits is associated with spatial variation in auxin accumulation and expression of a YUCCA biosynthesis gene
Source: Hortic Res. 2020 Sep 1;7:135. doi: 10.1038/s41438-020-00354-5 (PMC7459348; doi:10.1038/s41438-020-00354-5)
Supplement: Supplementary file 1 — Editorial Certificate [file 41438_2020_354_MOESM1_ESM.pdf]

美辑编译
源自美国 专注SCI服务

https://www.mjeditor.com

EDITORIAL CERTIFICATE

The English writing of the following manuscript was carefully edited by a native English speaker.

Manuscript information

ID：7096

Editing date：2020.06.10

Title：The curvature of cucumber fruit is associated with spatial variation in auxin accumulation and expression of a YUCCA biosynthetic gene.

Author：Shengnan Li, Chunhua Wang , Xiuyan Zhou, Dong Liu, Chunhong Liu, Jie Luan, Zhiwei Qin, Ming Xin.

Language writing before editing: ☐Very poor ☐Poor ☑Fair ☐Good ☐Very good ☐Excellent

Recommendation after language editing:
☐Submitting to target journal directly
☑Submitting to target journal after minor revision
☐Re-editing required after major revision
☐Not suitable for publication

Certificate by

Sophiya. K

Editor in Chief
MJ Language Editing Services, Shenzhen, China

MJ Language Editing Services, offers professional English language editing and publication support services to authors engaged in over 500 areas of research through its community of experienced editors, which includes doctors, published scientists, and researchers with peer review experience. Authors who work with MJ are guaranteed excellent language quality and timely delivery.

MJ Language Editing Services
Diwang Building, No. 5002 Shennan Road, Luohu District, Shenzhen, China
Tel:+086 0755 25100506
