# Supplementary material for: The curvature of cucumber fruits is associated with spatial variation in auxin accumulation and expression of a YUCCA biosynthesis gene
Source: Hortic Res. 2020 Sep 1;7:135. doi: 10.1038/s41438-020-00354-5 (PMC7459348; doi:10.1038/s41438-020-00354-5)
Supplement: Supplementary file 2 — Supplementary Information [file 41438_2020_354_MOESM2_ESM.docx]

**The curvature of cucumber fruit is associated with spatial variation in auxin accumulation and expression of a YUCCA biosynthetic gene**

**Running title:** *CsYUC10b* functions in curvature cucumber fruit.

Shengnan Li^1,3^, Chunhua Wang ^1,2,3^, Xiuyan Zhou^1^, Dong Liu^1^, Chunhong Liu^1^, Jie Luan^1^, Zhiwei Qin^1^, Ming Xin^1*^

^1^College of Horticulture and Landscape Architecture, Key Laboratory of Biology and Genetic Improvement of Horticultural Crops (Northeast Region), Northeast Agricultural University, Harbin 150030, China.

^2^College of Horticulture, Fujian Provincial Key Laboratory of Haixia Applied Plant Systems Biology, Fujian Agriculture and Forestry University, Fuzhou 350002, China

^3^These authors contributed equally

*Correspondence: Ming Xin (xinmingneau@126.com)

**Supplementary information**

TableS1 The number of fruits in L18, D0328-3 and D0859 genotype.

| **Genotype** | **Total plants** | **Fruit number**  **/per plant** | **Total fruits** | **Curved fruits** | **Straight**  **fruits** | **Curving ratios** |
| --- | --- | --- | --- | --- | --- | --- |
| L18 | 150 | 3-4 | 580 | 308 | 272 | 53.1% |
| D0328 | 50 | 4-5 | 220 | 86 | 134 | 39.1% |
| D0859 | 50 | 3-4 | 200 | 56 | 124 | 28.0% |

**TableS2** Sequences of the oligo nucleotides used in this study.

| **Oligo name** | **Oligo sequence（5’**→ **3’）** | **Description** | **Cucumber gene ID** |
| --- | --- | --- | --- |
| YUC10-F | TCCGCCCCAAACTACCTT | qRT-PCR analysis | Csa3G190380 |
| YUC10-R | CCGTCGCCACCACTAAAAA |  |  |
| LAX2-F | CATCTTTGGAGCCTGTTGTG | qRT-PCR analysis | Csa2G264590 |
| LAX2-R | ATGGGAGTGTCAGTGTTAGCA |  |  |
| AUX1-F | GACTTATATTTTTGGAGCTTGTTGT | qRT-PCR analysis | Csa3G731880 |
| AUX1-R | ATAGATGTATTTGAATTTCTGTGGC |  |  |
| GH3.1-F | CTACACATTGCTCTGCCACG | qRT-PCR analysis | Csa3G431430 |
| GH3.1-R | CCCTTCCACAAAATCCGC |  |  |
| CsYUC10b-F | ATGGCAGACACTACTGTGATAATCA | PCR clone | Csa3G190380 |
| CsYUC10b-R | TGATTATCACAGTAGTGTCTGCCAT |  |  |
| CsYUC10b-GF | CGCAAGCTTATGCATGGCAGACACTACT | Subcellular localization | Csa3G190380 |
| CsYUC10b-GR | GCCGGATCCTGATTATCACAGTAGTG |  |  |
| PCXSN-1250-F | CGGCAACAGGATTCAATCTTA | Transgenic detection |  |
| PCXSN-1250-R | CAAGCATTCTACTTCTATTGCAGC |  |  |

**TableS3** Obtain the high-quality clean reads

| **Sample** | **Raw data** | **Clean reads** |
| --- | --- | --- |
| C1 | 31589844 | 27074135(85.71%) |
| C2 | 20592406 | 16517441(80.21%) |

**TableS4** Functional analysis of differentially expressed genes (DEGs) on the C1 and C2 sides of curved fruit.

| **GO term** | **Ontology*** | **Description** | **Number of genes** | **%^＃^** | **p-value** | **Enrich-ment score** |
| --- | --- | --- | --- | --- | --- | --- |
| **Concave up-regulated** | | | | | | |
| GO:0007166 | P | Cell surface receptor-linked signal transduction | 48 | 2.5144 | 1.91E-14 | 9.78 |
| GO:0030528 | F | Transcription regulator activity | 213 | 11.1576 | 8.88E-07 | 6.09 |
| GO:0009873 | F | Ethylene-mediated signalling pathway | 33 | 1.7286 | 1.02E-05 | 5.90 |
| GO:0048316 | P | Seed development | 58 | 3.0382 | 3.55E-04 | 5.62 |
| GO:0007017 | P | Microtubule-based process | 27 | 1.4143 | 5.88E-06 | 5.43 |
| GO:0008361 | P | Regulation of cell size | 44 | 2.3048 | 2.39E-07 | 5.2 |
| GO:0009788 | P | Negative regulation of abscisic acid-mediated signalling | 5 | 0.2619 | 0.0683081 | 3.54 |
| GO:0048469 | P | Cell maturation | 11 | 0.5762 | 6.83E-04 | 3.48 |
| GO:0009913 | P | Epidermal cell differentiation | 26 | 1.3619 | 5.11E-06 | 3.41 |
| GO:0008360 | P | Regulation of cell shape | 6 | 0.3143 | 8.67E-04 | 2.72 |
| GO:0016567 | P | Protein ubiquitination | 17 | 0.8905 | 0.0565228 | 2.56 |
| GO:0043167 | F | Ion binding | 322 | 16.8674 | 0.0211072 | 2.36 |
| GO:0046527 | F | Glucosyltransfer-ase activity | 26 | 1.3619 | 1.07E-04 | 2.34 |
| GO:0009926 | P | Auxin polar transport | 10 | 0.5762 | 0.0040169 | 1.93 |
| **Convex up-regulated** | | | | | | |
| GO:0015979 | P | Photosynthesis | 24 | 1.5335 | 2.09E-04 | 6.68 |
| GO:0003735 | F | Structural constituent of ribosome | 48 | 3.0670 | 2.39E-05 | 4.46 |
| GO:0009853 | P | Photorespiration | 9 | 0.5750 | 0.002573 | 3.59 |
| GO:0031974 | C | Membrane-enclosed lumen | 62 | 3.9616 | 2.11E-04 | 2.83 |
| GO:0016168 | F | Chlorophyll binding | 9 | 0.5750 | 1.43E-04 | 2.66 |
| GO:0009941 | C | Chloroplast envelope | 34 | 2.1725 | 0.071905 | 2.55 |
| GO:0009416 | P | Response to light stimulus | 41 | 2.6198 | 0.009019 | 2.46 |
| GO:0009767 | P | Photosynthetic electron transport chain | 5 | 0.3194 | 0.085335 | 2.22 |
| GO:0022900 | P | Electron transport chain | 25 | 1.5974 | 6.52E-05 | 2.13 |
| GO:0006260 | P | DNA replication | 15 | 0.9584 | 0.0122 | 1.93 |
| GO:0016407 | F | Acetyltransferase activity | 8 | 0.5111 | 0.091764 | 1.43 |
| GO:0009955 | F | Adaxial/abaxial pattern formation | 6 | 0.3833 | 0.011129 | 1.37 |
| GO:0055114 | P | Oxidation reduction | 110 | 7.0287 | 8.22E-07 | 1.37 |
| GO:0009733 | P | Response to auxin stimulus | 31 | 1.9808 | 0.013738 | 1.31 |
| GO:0009744 | P | Response to sucrose stimulus | 7 | 0.4472 | 0.016651 | 1.31 |

*Biological Process (P) and Molecular Function (F). ^＃^The percentage of genes in each category accounted for the total DEGs (total DEGs were homologous to Arabidopsis thaliana, and repeated genes were removed).

**Table S5** Number of DEGs associated with plant hormones.

| **C1 to C2** | **Total number** | **Ethylene** | **IAA** | **CK** | **ABA** | **GA** |
| --- | --- | --- | --- | --- | --- | --- |
| up | 62 | 31 | 13 | 8 | 6 | 4 |
| down | 18 | 9 | 8 | 1 | 0 | 0 |
| total | 80 | 40 | 21 | 9 | 6 | 4 |

| **Genotype** | **Total plants** | **Fruit number**  **/per plant** | **Total fruits** | **Curved fruits** | **Straight**  **fruits** | **Curving ratios** |
| --- | --- | --- | --- | --- | --- | --- |
| L18(control) | 50 | 3-4 | 188 | 99 | 89 | 52.6% |
| OX4 | 50 | 3-4 | 182 | 39 | 143 | 21.5% |
| OX7 | 50 | 3-4 | 189 | 39 | 150 | 20.6% |
| OX10 | 50 | 3-4 | 176 | 35 | 141 | 19.9% |

**Table S6** The number of fruits in control, OX4, OX7 and OX10.

**Figure legends**

**Fig. S1.** The transcriptome quality and completeness of curved fruit between concave (C1) convex (C2) sides at 2 DPA. (a) Quality analysis. (b) Sequence content distribution. (c) Length distribution of clean reads.

**Fig. S2.** Differentially expressed genes between the concave (C1) and convex (C2) sides; the top red indicates up-regulated genes and the bottom red indicates down-regulated genes.

**Fig. S3.** Angle of curvature in cucumber. (a) Angle of curvature in the curved (45° and 70-90°) and straight fruits at 2, 4, 6, 8, 10, 12, 14, 16 and 18 DPA. (b) Fruit shape at 2, 4, 6, 8 and 10 DPA.

**Fig. S4.** Fruit phenotype between control and curved fruits treated with 0.1µM AVG at 0 and 4 DPA.

**Fig. S5.** Sequence alignment and phylogenetic analysis of CsYUC10b. (a) Sequence analysis of CsYUC10b. (b) Phylogenetic analysis of CsYUC10b with other YUC proteins by neighbor-joining algorithm. *Cs*, *Cucumis sativus*; *At*, *Arabidopsis thaliana*; *Os*, *Oryza sativa*; *Zm*, *Zea mays*; *Mn*, *Morus*; *Cm*, *Cucumis melo*; *Tc*, *Theobrom*a. (c) The conserved FAD binding component and FMO identifying sequence.

**Fig. S6.** Subcellular localization of CsYUC10b protein in *Arabidopsis* protoplasts. Green fluorescence indicates localization of fusion protein. Bar = 10μm.

**Fig. S7.** Identification of overexpressed-*CsYUC10b* cucumber plants by PCR. 1-44, electrophoretic band with correct position in the overexpressed-*CsYUC10b* cucumber plants. M, DNA DL2000; +, positive control (p-1250); -, water control.

**Fig. S8.** The microstructure of cells in the exocarp at 100× magnification at 6 DPA. (a) Concave side of curved fruit. (b) Convex side of curved fruit.

Fig.S1


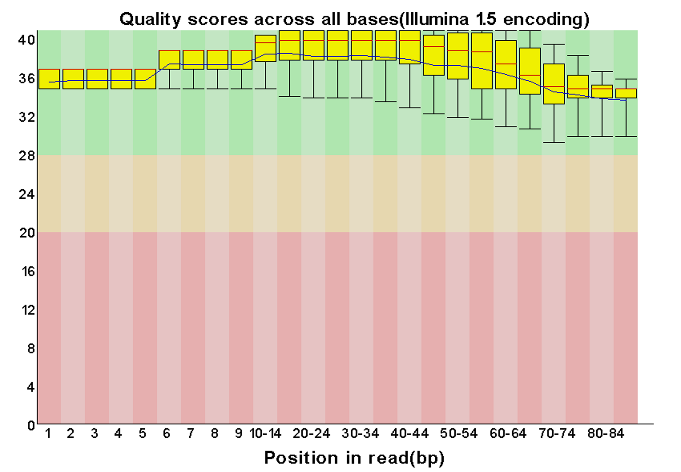

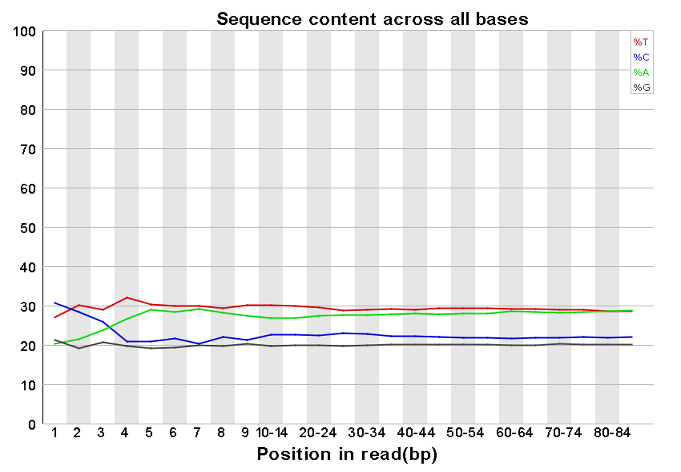

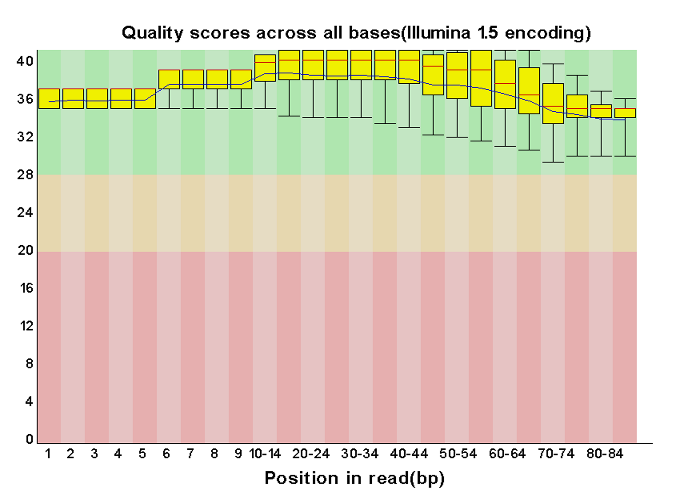

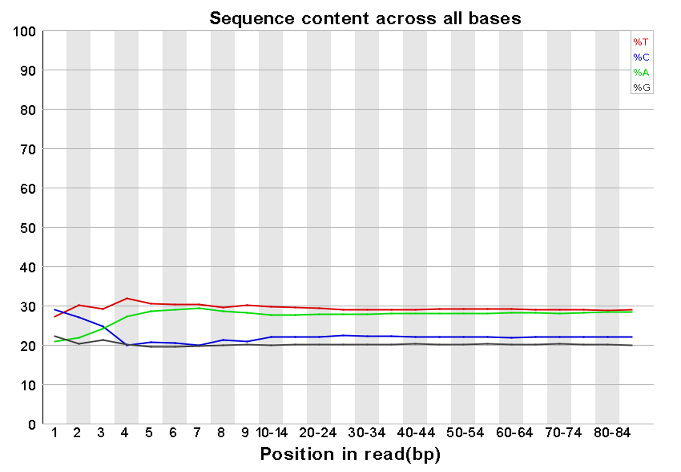


C1

C1

C2

C2


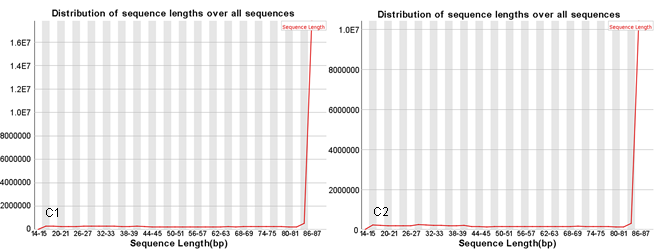


Fig.S2


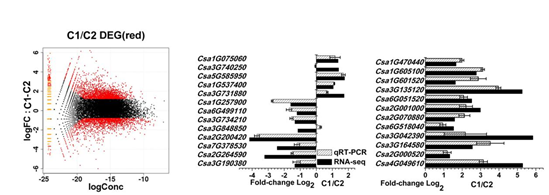


Fig.S3


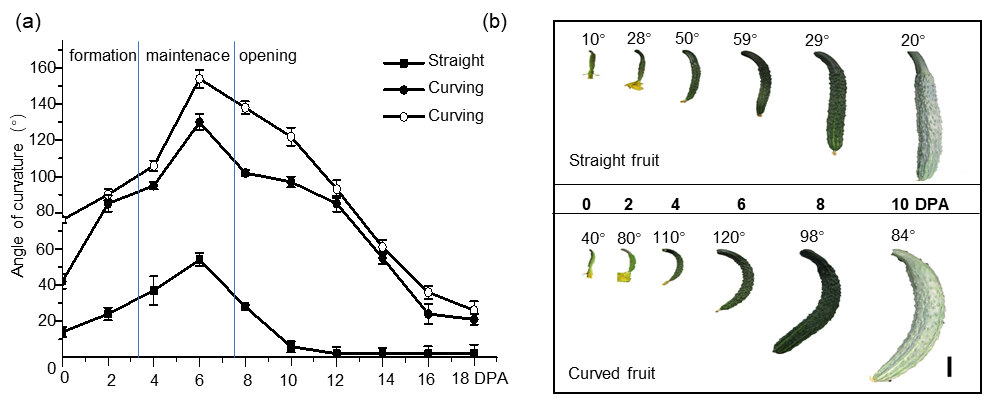


Fig.S4


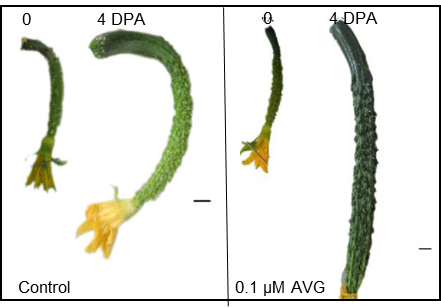


Fig.S5


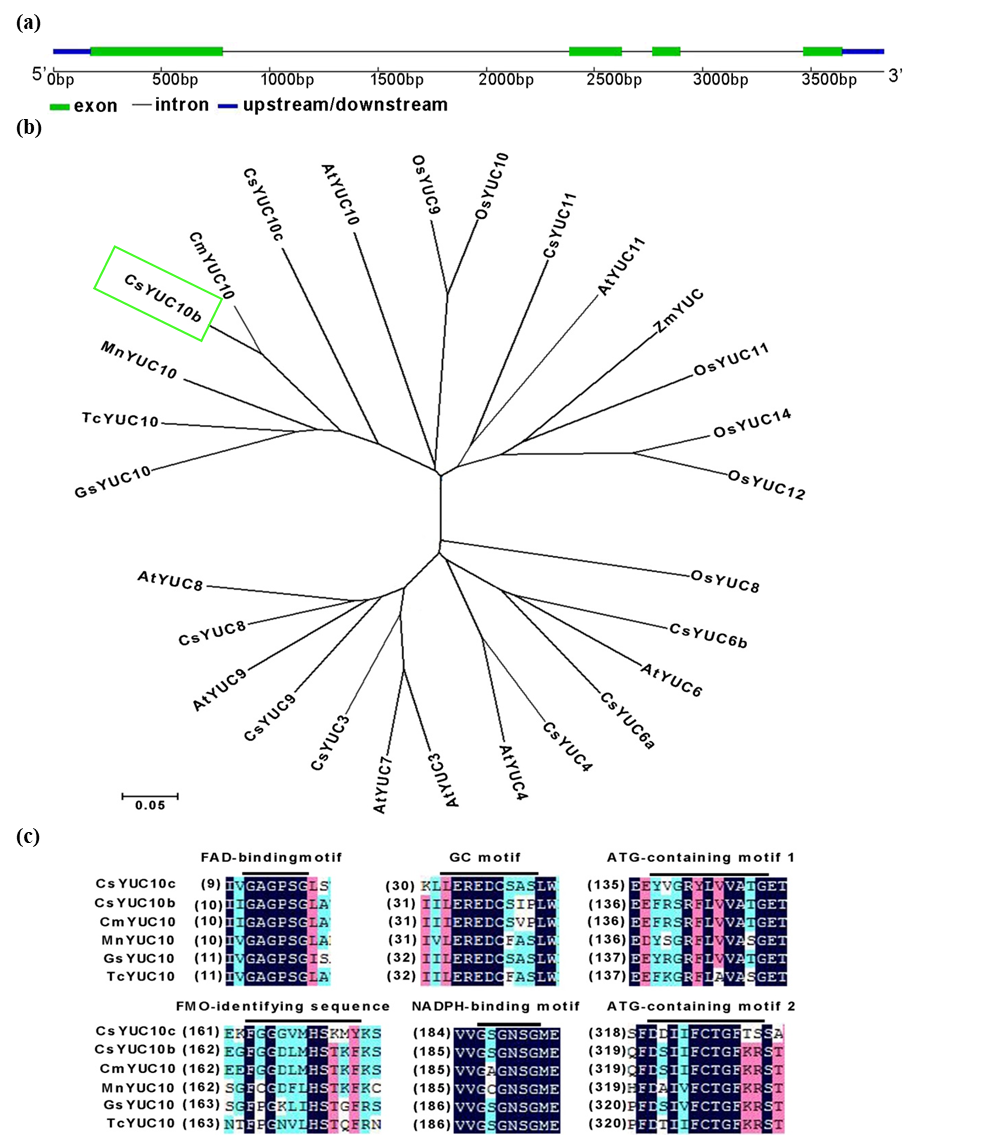


Fig.S6


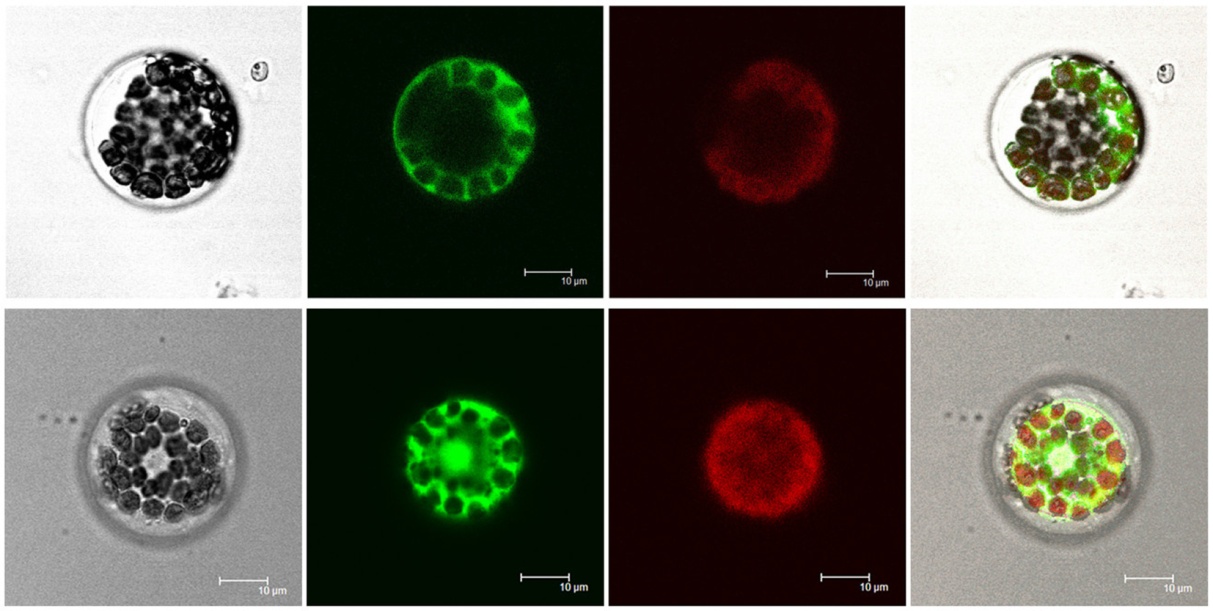


35S:GFP

35S:CsYUC10b-GFP

Bright

GFP

ChloropyII

Merged

Fig.S7


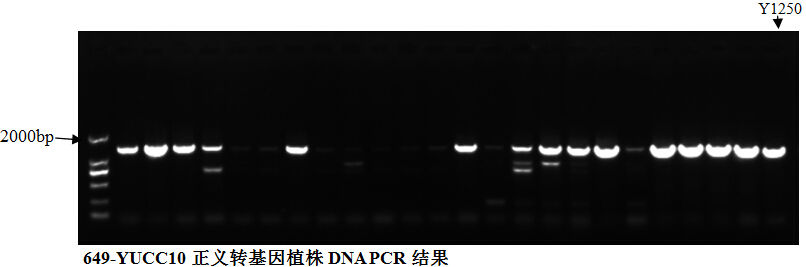


M 1 2 3 4 5 6 7 8 9 10 11 12 13 14 15 16 17 18 19 20 21 22 23 24

2000 bp

2000 bp


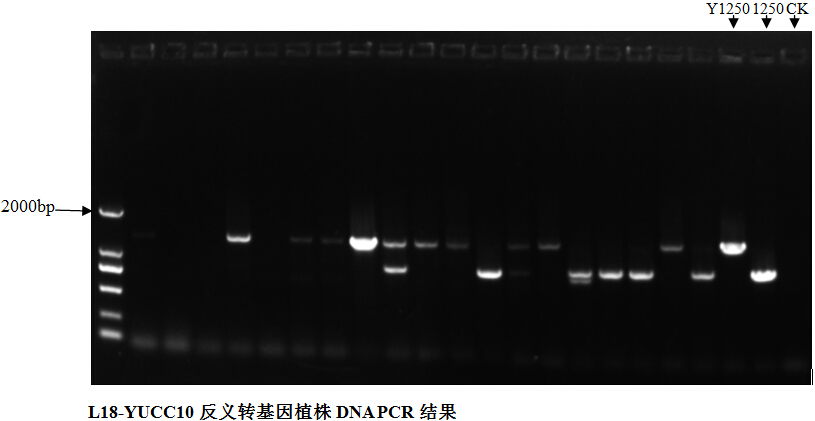


M 25 26 27 28 29 30 31 32 33 34 35 36 37 38 39 40 41 42 43 44 + -

Fig.S8


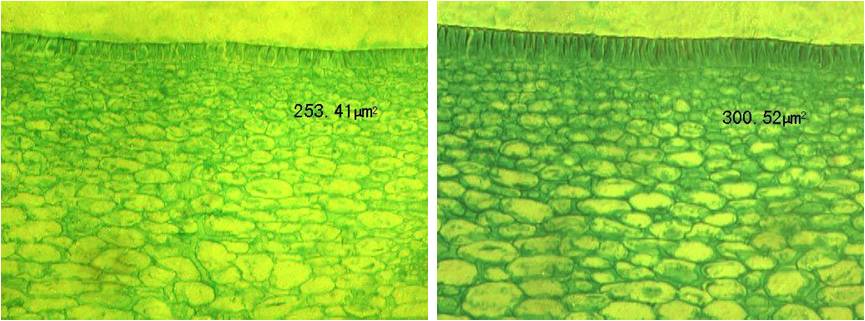


Convex

Concave
